# Editorial: Preclinical models and emerging technologies to study the effects of the tumor microenvironment on cancer heterogeneity and drug resistance

**DOI:** 10.3389/fonc.2023.1289756

**Published:** 2023-09-28

**Authors:** Giulia Adriani, Paola Cappello, Sara Lovisa

**Affiliations:** ^1^ Singapore Immunology Network (SIgN), Agency for Science, Technology and Research (A*STAR), Singapore, Singapore; ^2^ Department of Biomedical Engineering, National University of Singapore (NUS), Singapore, Singapore; ^3^ Department of Molecular Biotechnology and Health Sciences, University of Turin, Torino, Italy; ^4^ Department of Biomedical Sciences, Humanitas University, Milan, Italy; ^5^ Department of Gastroenterology, IRCCS Humanitas Research Hospital, Rozzano, Milan, Italy

**Keywords:** tumor microenvironment, tumor heterogeneity, scRNA seq, spatial biology, preclinical tumor models, 3D tumor models, *in vivo* tumor models

Cancer research has witnessed remarkable advancements in recent years, with molecularly targeted treatments and immunotherapy revolutionizing patient care. However, some cancer types, such as pancreatic ductal adenocarcinoma, continue to defy treatment, and the emergence of drug resistance remains a significant challenge, hampering efforts to achieve lasting remission, especially in metastatic disease. In the past, cancer development was solely attributed to the appearance of genomic alterations in cancer cells. In the last decades, a broad consensus arose about the necessity for cancer cells to cooperate with host cells, such as fibroblasts, vascular cells, lymphatic cells, and immune cells during carcinogenesis and cancer progression. Therefore, the focus shifted toward understanding the tumor microenvironment (TME) heterogeneity and its impact on cancer evolution and drug resistance. In this dynamic landscape, innovative preclinical models and cutting-edge technologies have emerged as indispensable tools in TME characterization and, in general, in the efforts against cancer (**Figure 1**).

**Figure 1:**
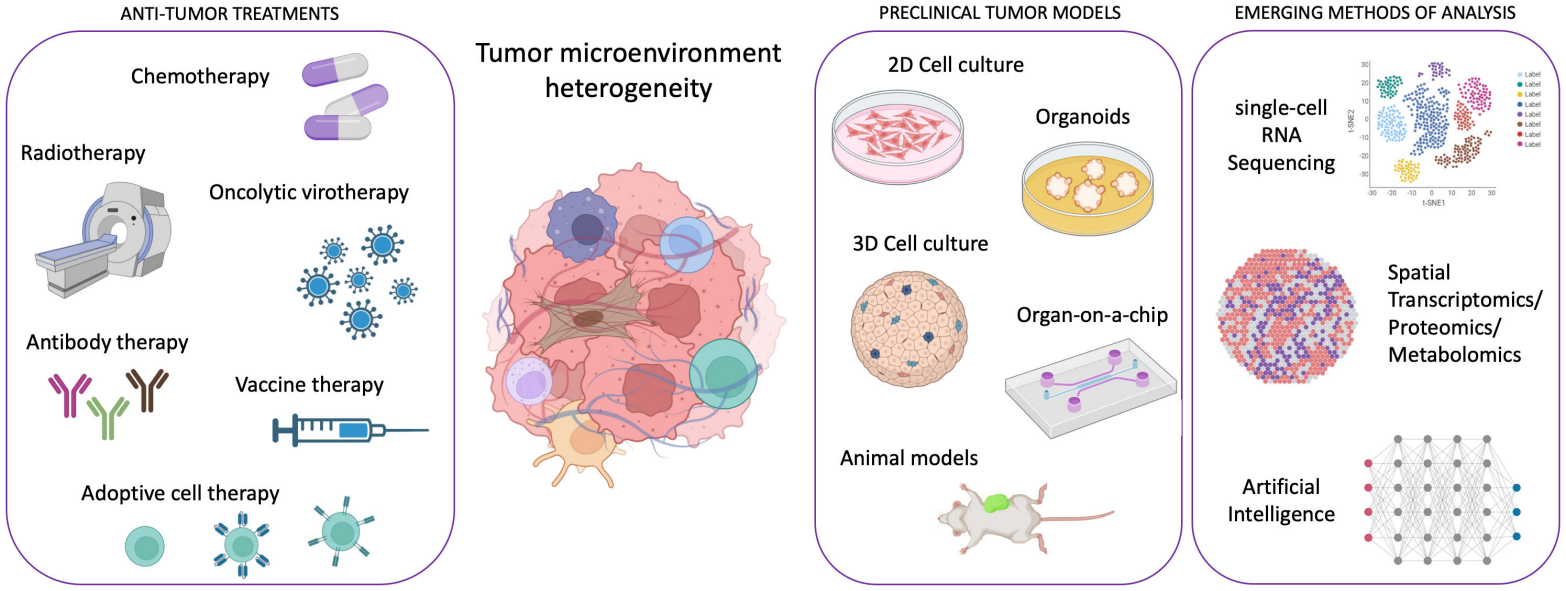
Scheme illustrating the framework of emergent technologies and preclinical tumor models for addressing the complexity of tumor microenvironment heterogeneity in order to enhance the efficacy of anti-tumor therapies. Created with Biorender.com.

Understanding the heterogeneous nature of tumors has presented significant challenges, but recent insights into the TME role have lightened up new paths for therapeutic exploration. This Research Topic examine various facets of the TME influence on cancer heterogeneity and drug resistance, encompassing diverse cancer types, including breast, colorectal, pancreatic, liver, and head and neck cancers. While each article offers a unique perspective, they collectively emphasize the role of the TME in shaping tumor behavior and response to treatment.

Genta et al., Huang and Tu, and Proietto et al. highlight the advances of preclinical models in deciphering the complexities of the TME. Genetically engineered mouse models, patient-derived organoids and xenografts, and three-dimensional (3D) cell cultures have all emerged as essential tools to study cancer progression, acquired heterogeneity, and predict drug efficacy with an enhanced clinical relevance. Genta et al. encompass both *in vitro* and *in vivo* platforms for drug testing, stressing the relevance of patient-derived models to maintain the molecular affinity with the parental tumors and predict patient response. However, the authors also draw attention to the challenge that, despite their use in co-clinical trials and their value in understanding drug resistance, the data generated often arrive too late to modify the therapeutic plans effectively. Huang and Tu dive into the specific challenges posed by tumor heterogeneity and the microenvironment in vascularized tumor models, widely discussing the advantages and limitations of organ-on-a-chip models in replicating the vascularized tumor microenvironment. The addition of different types of supporting cells, namely pericytes, astrocytes, or lymphatic cells, makes those organ-on-a-chip models closely mimic the heterotypic cellular interactions within tumors. Further, the inclusion of organ-specific normal cells allows for assessing general drug toxicity. However, the authors also comment on some limitations, such as the accessibility to quantify cellular forces and cell stiffness. Proietto et al. extensively review the TME preclinical models and broaden the discussion to include the role of emerging single-cell, spatial genomic, and metabolomic technologies in understanding tumor complexity and evolution to design better targeted therapies. The authors highlight the need for multidisciplinary approaches to combat the challenges posed by tumor heterogeneity. Lee et al. remarkably contribute to further highlight the growing significance of cutting-edge spatial omics technologies in dissecting TME heterogeneity, also introducing the value added by artificial intelligence in unraveling the complex interactions within the TME.

The other articles in the Research Topic offer innovative insights on specific cancers, discussing advances in their modeling for preclinical studies and their organotypic TME role in drug resistance. Yau et al. look at the importance of incorporating diverse cell populations within colorectal cancer spheroid models to mimic the intricate signaling pathways and heterogeneity found *in vivo*. Salemme et al. extensively discuss the characteristics of breast cancer, a highly heterogeneous disease, that leads to drug resistance and encompass the advances in developing *in vivo* and *in vitro* models to recapitulate breast cancer complexity. Another work about breast cancer by Lamouline et al. focuses on *in vitro* models for breast cancer metastasis in bone, elegantly discussing the cellular heterogeneity of this specific microenvironment, shedding light on the critical need to account for the different microenvironments in *in vitro* models dedicated to the study of metastasis to accurately mimic tissue-specific cell-cell and cell-extracellular matrix (ECM) interactions when assessing drug sensitivity. Indeed, van Tienderen et al. present a novel *in vitro* model of cholangiocarcinoma metastasis by ingeniously combining patient-derived organoids with decellularized human tissues, confirming the intricate interplay between cancer cells and the ECM in driving dissemination to distant organs such as lung and lymph nodes. Dedicated to studying drug pharmacological effects and tissue physiology is the model proposed by Greier et al. in their paper, in which they demonstrate the promising potential of 3D primary slice cultures to provide preclinical models for studying head and neck cancer and obtain data in a week frame to guide clinicians in the choice of treatments. Giustarini et al. establish human 3D heterocellular tumor spheroids as models mirroring the complexity of the pancreatic ductal adenocarcinoma environment. Notably, these tissue-specific spheroids reveal immunosuppressive tendencies and mimic spatial and cytokine signatures observed in patients, confirming their value for better therapy testing. The more we advance our ability to reproduce complex high-throughput systems *in vitro*, particularly through the consistent and standardized utilization of patient-derived samples for drug library testing, the sooner we will have a time-saving and cost-effective alternative to *in vivo* mouse models. While very powerful and extensively used, these animal models present ethical and variance concerns in rigorously reflecting human tumor evolution and heterogeneity, delaying drug development processes.

This Research Topic presents a comprehensive picture of the recent advancements in understanding the TME influence on cancer heterogeneity and drug resistance. As these studies collectively suggest, it is clear that an accurate understanding of the TME role is pivotal for designing effective cancer therapies. This Research Topic highlights how the essential collaboration between clinicians, researchers, and technologists in developing advanced preclinical models and cutting-edge technologies is uncovering the subtle interactions between cancer cells, immune cells, and the surrounding microenvironment to develop effective therapeutics that will tackle drug resistance and improve patient outcomes worldwide.

## Author contributions

GA: Writing – original draft. PC: Writing – review & editing. SL: Writing – review & editing.

